# *rac*-7,7′,9,9′-Tetra­phenyl-9a,9a′-bi(7,8,9,9a-tetra­hydro-6a*H*-penta­leno[1,2,3-*ij*]naphthalen-8-one)

**DOI:** 10.1107/S1600536812014146

**Published:** 2012-04-06

**Authors:** Xiangdong Zhang, Junwei Ye, Weitao Gong, Yuan Lin, Guiling Ning

**Affiliations:** a State Key Laboratory of Fine Chemicals and School of Chemical Engineering, Dalian University of Technology, Dalian 116012, People’s Republic of China

## Abstract

The racemic title compound, C_54_H_38_O_2_, consists of two C-linked penta­leno[1,2,3-*ij*]naphthalenone moieties, the crowded aryl ring substitution on the cyclo­pentane rings forcing the two segments to assume a conformation which has pseudo-twofold rotational symmetry, with a dihedral angle between the naphthalene substituent groups of 55.30 (8)°. In each segment, the two phenyl rings have different conformational orientations, with inter-ring dihedral angles of 34.7 (2) and 49.63 (16)°. Each cyclo­pentane ring has the same relative configuration in its four chiral centres and together with the fused naphthalene ring assumes an overall chair-like conformation.

## Related literature

For photoluminescence properties of naphthalene compounds, see: Cai *et al.* (2010 ▶); Haneline *et al.* (2002 ▶); Koning *et al.* (2003 ▶); Tsubaki *et al.* (2006 ▶). For a related structure, see: Dyker (1993 ▶).
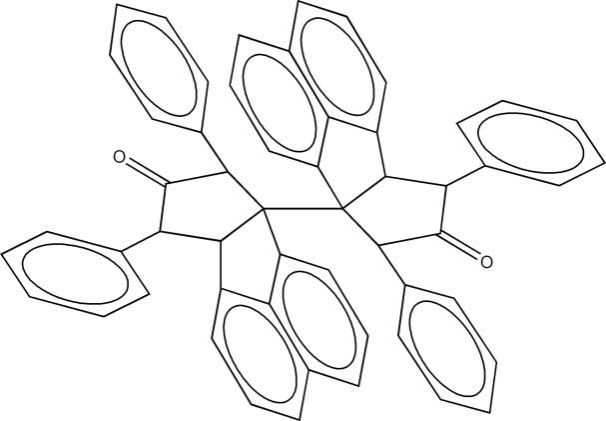


## Experimental

### 

#### Crystal data

C_54_H_38_O_2_
*M* *_r_* = 718.84
Monoclinic, 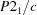

*a* = 14.325 (3) Å
*b* = 13.448 (3) Å
*c* = 19.914 (4) Å
β = 101.449 (5)°
*V* = 3759.9 (14) Å^3^
*Z* = 4
Mo *K*α radiation
μ = 0.08 mm^−1^
*T* = 275 K
0.23 × 0.22 × 0.19 mm

#### Data collection

Bruker CCD area-detector diffractometer
Absorption correction: multi-scan (*SADABS*; Sheldrick, 1996 ▶) *T*_min_ = 0.983, *T*_max_ = 0.986
42062 measured reflections
8342 independent reflections
4812 reflections with *I* > 2σ(*I*)
*R*_int_ = 0.051

#### Refinement

*R*[*F* ^2^ > 2σ(*F* ^2^)] = 0.066
*wR*(*F* ^2^) = 0.161
*S* = 1.05
8342 reflections
505 parameters
H-atom parameters constrained
Δρ_max_ = 0.21 e Å^−3^
Δρ_min_ = −0.20 e Å^−3^

### 

Data collection: *SMART* (Bruker, 1997 ▶); cell refinement: *SAINT* (Bruker, 1997 ▶); data reduction: *SAINT*; program(s) used to solve structure: *SHELXS97* (Sheldrick, 2008 ▶); program(s) used to refine structure: *SHELXL97* (Sheldrick, 2008 ▶); molecular graphics: *SHELXTL* (Sheldrick, 2008 ▶); software used to prepare material for publication: *SHELXL97*.

## Supplementary Material

Crystal structure: contains datablock(s) global, I. DOI: 10.1107/S1600536812014146/zs2187sup1.cif

Structure factors: contains datablock(s) I. DOI: 10.1107/S1600536812014146/zs2187Isup2.hkl

Additional supplementary materials:  crystallographic information; 3D view; checkCIF report

## Figures and Tables

**Fig. 1.**
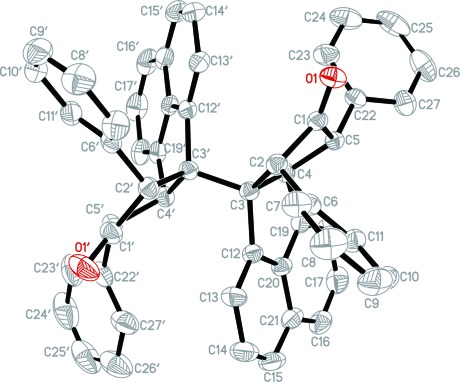
The molecular conformation and atom numbering scheme for the title compound, showing 30% probability displacement ellipsoids.

**Fig. 2.**
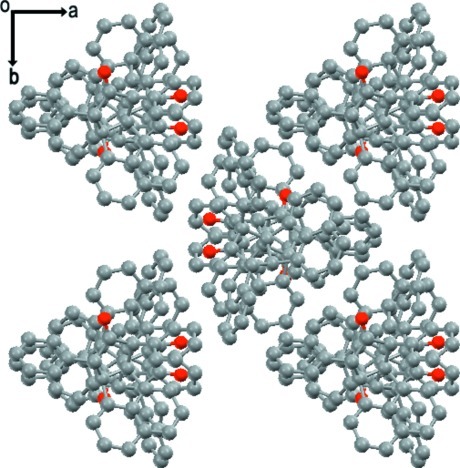
The stacking mode of the title compound.

**Fig. 3.**
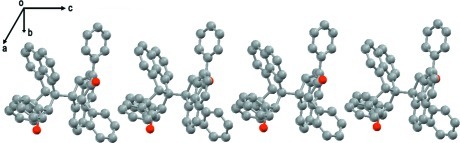
A view of the molecular stacks of the title compound extending along the c axis.

## supplementary crystallographic information

### Comment

Naphthalene derivatives have attracted considerable attention because of their superb photoluminescence (PL) performance (Cai *et al.*, 2010; Haneline *et al.*, 2002; Koning *et al.*, 2003; Tsubaki *et al.*, 2006). The investigation of structures of nonplanar naphthalene derivatives therefore constitutes a significant thrust in PL materials research. In the present study, a naphthalene and phenyl substituted cyclopentanone compound C_54_H_38_O_2_ has been synthesized and its crystal structure is reported here. A similar structure with half the skeleton of the title compound has been reported (Dyker, 1993).

The racemic title compound (Fig. 1) consists of two C-linked 2,5-diphenyl-3,4-naphthylcyclopentan-1-one moieties, the linking bond [C3—C3' = 1.578 (3) Å] being elongated due to the steric crowding afforded by the aryl ring substituents on the cyclopentane rings. The two segments assume a conformation which gives the molecule pseudo–twofold rotational symmetry, with a dihedral angle between the naphthalene groups of 55.30 (8)°. The torsion angle C4—C3—C3'—C4' about the C—C bridge is -63.8 (2)°. In each segment, the two phenyl rings have different conformational orientations, with inter-ring dihedral angles of 34.7 (2) and 49.63 (16)°. Each cyclopentane ring has the same relative configuration in its four chiral centres [C2(*S*, C3(*S*, C4(*S*, C5(*R* and C2'(*S*, C3'(*S*, C4'(*S*, C5'(*R*] and together with the naphthalene ring give an overall chair-like conformation. As expected there are no significant intermolecular interactions, giving simple unassociated molecular stacks (Figs. 2 and 3).

### Experimental

The title compound was prepared by a coupling reaction with 2,5-diphenyl-3-hydroxyl-3,4-naphthocyclopentan-1-one. This ketone (0.38 g, 1 mmol) and Me_3_SiCl (0.61 g, 6 mmol) were dissolved in 10 ml of dry toluene and a suspension of 0.9 g (6 mmol) of NaI in 1 ml of anhydrous CH_3_CN was added. The system was stirred at room temperature with light-avoidance for 8 h. When the reactant was exhausted, the reaction was cooled to 273 K and quenched with 10 ml of water. The mixture was extracted with diethyl ether, and the organic fraction was washed with aqueous sodium thiosulfate and brine in sequence, and evaporated under reduced pressure to give the crude product which was purified by flash chromatography (eluent: dichloromethane/petroleum ether, 1:5) to give title compound (0.23 g). Colorless crystals were obtained from a dichloromethane solution layered with methanol after a few days.

### Refinement

The H atoms were placed in calculated positions [C—H = 0.93 Å (aromatic) or 0.98 Å (methine) and refined in the riding-model approximation with *U*_iso_(H) = 1.2*U*_eq_(C).

### Crystal data

**Table:**

C_54_H_38_O_2_	*F*(000) = 1512
*M**_r_* = 718.84	*D*_x_ = 1.270 Mg m^−^^3^
Monoclinic, *P*2_1_/*c*	Mo *K*α radiation, λ = 0.71073 Å
Hall symbol: -P 2ybc	Cell parameters from 42062 reflections
*a* = 14.325 (3) Å	θ = 2.6–27.7°
*b* = 13.448 (3) Å	µ = 0.08 mm^−^^1^
*c* = 19.914 (4) Å	*T* = 275 K
β = 101.449 (5)°	Block, colorless
*V* = 3759.9 (14) Å^3^	0.23 × 0.22 × 0.19 mm
*Z* = 4	

### Data collection

**Table:**

Bruker CCD area-detector diffractometer	8342 independent reflections
Radiation source: fine-focus sealed tube	4812 reflections with *I* > 2σ(*I*)
Graphite monochromator	*R*_int_ = 0.051
φ and ω scans	θ_max_ = 27.7°, θ_min_ = 2.6°
Absorption correction: multi-scan (*SADABS*; Sheldrick, 1996)	*h* = −18→18
*T*_min_ = 0.983, *T*_max_ = 0.986	*k* = −15→17
42062 measured reflections	*l* = −25→22

### Refinement

**Table:**

Refinement on *F*^2^	Primary atom site location: structure-invariant direct methods
Least-squares matrix: full	Secondary atom site location: difference Fourier map
*R*[*F*^2^ > 2σ(*F*^2^)] = 0.066	Hydrogen site location: inferred from neighbouring sites
*wR*(*F*^2^) = 0.161	H-atom parameters constrained
*S* = 1.05	*w* = 1/[σ^2^(*F*_o_^2^) + (0.0377*P*)^2^ + 2.9173*P*] where *P* = (*F*_o_^2^ + 2*F*_c_^2^)/3
8342 reflections	(Δ/σ)_max_ < 0.001
505 parameters	Δρ_max_ = 0.21 e Å^−^^3^
0 restraints	Δρ_min_ = −0.20 e Å^−^^3^

### Special details

**Table:**

Geometry. All e.s.d.'s (except the e.s.d. in the dihedral angle between two l.s. planes) are estimated using the full covariance matrix. The cell e.s.d.'s are taken into account individually in the estimation of e.s.d.'s in distances, angles and torsion angles; correlations between e.s.d.'s in cell parameters are only used when they are defined by crystal symmetry. An approximate (isotropic) treatment of cell e.s.d.'s is used for estimating e.s.d.'s involving l.s. planes.
Refinement. Refinement of *F*^2^ against ALL reflections. The weighted *R*-factor *wR* and goodness of fit *S* are based on *F*^2^, conventional *R*-factors *R* are based on *F*, with *F* set to zero for negative *F*^2^. The threshold expression of *F*^2^ > σ(*F*^2^) is used only for calculating *R*-factors(gt) *etc*. and is not relevant to the choice of reflections for refinement. *R*-factors based on *F*^2^ are statistically about twice as large as those based on *F*, and *R*- factors based on ALL data will be even larger.

### Fractional atomic coordinates and isotropic or equivalent isotropic displacement parameters (Å^2^)

**Table:**

	*x*	*y*	*z*	*U*_iso_*/*U*_eq_	
O1	0.44413 (13)	0.31609 (14)	0.25183 (10)	0.0548 (5)	
O1'	0.14712 (15)	0.08762 (17)	−0.05330 (12)	0.0808 (7)	
C1	0.36038 (18)	0.30516 (18)	0.22892 (12)	0.0412 (6)	
C1'	0.16606 (19)	0.1682 (2)	−0.02781 (15)	0.0553 (7)	
C2	0.31861 (17)	0.22012 (18)	0.18147 (12)	0.0424 (6)	
H2	0.3620	0.2062	0.1503	0.051*	
C2'	0.25646 (18)	0.18987 (19)	0.02529 (13)	0.0458 (6)	
H2'	0.2660	0.1355	0.0587	0.055*	
C3	0.22333 (16)	0.26340 (18)	0.13837 (12)	0.0388 (6)	
C3'	0.23484 (17)	0.28710 (18)	0.06285 (12)	0.0402 (6)	
C4	0.20277 (17)	0.36262 (18)	0.17678 (12)	0.0400 (6)	
H4	0.2057	0.4205	0.1474	0.048*	
C4'	0.13773 (18)	0.32998 (19)	0.01889 (12)	0.0444 (6)	
H4'	0.0882	0.3299	0.0465	0.053*	
C5	0.28023 (17)	0.37074 (18)	0.24304 (12)	0.0408 (6)	
H5	0.2544	0.3381	0.2794	0.049*	
C5'	0.10759 (19)	0.2619 (2)	−0.04458 (14)	0.0549 (7)	
H5'	0.1307	0.2942	−0.0823	0.066*	
C6	0.31185 (18)	0.12745 (18)	0.22407 (13)	0.0423 (6)	
C6'	0.34115 (18)	0.1922 (2)	−0.00976 (13)	0.0463 (6)	
C7	0.3683 (2)	0.0463 (2)	0.21658 (16)	0.0584 (7)	
H7	0.4090	0.0493	0.1856	0.070*	
C7'	0.4174 (2)	0.1298 (2)	0.01212 (16)	0.0647 (8)	
H7'	0.4168	0.0868	0.0486	0.078*	
C8	0.3647 (2)	−0.0398 (2)	0.2549 (2)	0.0776 (10)	
H8	0.4020	−0.0944	0.2488	0.093*	
C8'	0.4955 (2)	0.1306 (3)	−0.0199 (2)	0.0807 (10)	
H8'	0.5467	0.0884	−0.0044	0.097*	
C9	0.3061 (2)	−0.0439 (3)	0.30166 (19)	0.0764 (10)	
H9	0.3040	−0.1013	0.3274	0.092*	
C9'	0.4979 (2)	0.1925 (3)	−0.0738 (2)	0.0768 (10)	
H9'	0.5499	0.1921	−0.0953	0.092*	
C10	0.2513 (2)	0.0357 (2)	0.31033 (15)	0.0602 (8)	
H10	0.2119	0.0328	0.3422	0.072*	
C10'	0.4226 (2)	0.2553 (3)	−0.09590 (15)	0.0642 (8)	
H10'	0.4237	0.2982	−0.1323	0.077*	
C11	0.2539 (2)	0.1205 (2)	0.27215 (13)	0.0510 (7)	
H11	0.2160	0.1744	0.2787	0.061*	
C11'	0.3453 (2)	0.2549 (2)	−0.06445 (13)	0.0549 (7)	
H11'	0.2947	0.2977	−0.0802	0.066*	
C12	0.13540 (17)	0.19949 (19)	0.13976 (12)	0.0431 (6)	
C12'	0.30568 (18)	0.37184 (19)	0.06057 (12)	0.0428 (6)	
C13	0.1127 (2)	0.1028 (2)	0.12165 (15)	0.0569 (7)	
H13	0.1555	0.0628	0.1045	0.068*	
C13'	0.40160 (19)	0.3802 (2)	0.08404 (13)	0.0501 (7)	
H13'	0.4362	0.3274	0.1067	0.060*	
C14	0.0239 (2)	0.0648 (3)	0.12933 (17)	0.0703 (9)	
H14	0.0086	−0.0004	0.1160	0.084*	
C14'	0.4475 (2)	0.4701 (2)	0.07354 (15)	0.0624 (8)	
H14'	0.5128	0.4754	0.0896	0.075*	
C15	−0.0401 (2)	0.1193 (3)	0.15528 (16)	0.0685 (9)	
H15	−0.0984	0.0915	0.1587	0.082*	
C15'	0.3997 (2)	0.5494 (2)	0.04072 (16)	0.0661 (9)	
H15'	0.4323	0.6077	0.0354	0.079*	
C16	−0.0753 (2)	0.2827 (3)	0.20793 (16)	0.0697 (9)	
H16	−0.1352	0.2624	0.2138	0.084*	
C16'	0.2416 (3)	0.6173 (2)	−0.02160 (16)	0.0712 (9)	
H16'	0.2672	0.6786	−0.0299	0.085*	
C17	−0.0424 (2)	0.3745 (3)	0.22916 (16)	0.0728 (10)	
H17	−0.0803	0.4154	0.2503	0.087*	
C17'	0.1474 (3)	0.5996 (2)	−0.04458 (16)	0.0733 (10)	
H17'	0.1098	0.6497	−0.0681	0.088*	
C18	0.0470 (2)	0.4105 (2)	0.22040 (14)	0.0599 (8)	
H18	0.0673	0.4739	0.2351	0.072*	
C18'	0.1045 (2)	0.5084 (2)	−0.03424 (14)	0.0624 (8)	
H18'	0.0400	0.4979	−0.0512	0.075*	
C19	0.10300 (17)	0.3502 (2)	0.18994 (13)	0.0453 (6)	
C19'	0.15998 (19)	0.4353 (2)	0.00136 (12)	0.0477 (6)	
C20	−0.01890 (19)	0.2181 (2)	0.17719 (14)	0.0567 (8)	
C20'	0.3014 (2)	0.5432 (2)	0.01489 (14)	0.0567 (7)	
C21	0.07006 (17)	0.2551 (2)	0.16821 (13)	0.0451 (6)	
C21'	0.25666 (19)	0.45336 (19)	0.02589 (12)	0.0444 (6)	
C22	0.30952 (17)	0.47445 (19)	0.26837 (13)	0.0458 (6)	
C22'	0.0013 (2)	0.2486 (3)	−0.06871 (15)	0.0640 (8)	
C23	0.3551 (2)	0.5379 (2)	0.23124 (17)	0.0648 (8)	
H23	0.3667	0.5175	0.1891	0.078*	
C23'	−0.0474 (3)	0.3071 (3)	−0.12167 (17)	0.0907 (12)	
H23'	−0.0141	0.3523	−0.1433	0.109*	
C24	0.3837 (2)	0.6309 (2)	0.2554 (2)	0.0819 (11)	
H24	0.4149	0.6724	0.2296	0.098*	
C24'	−0.1452 (3)	0.2986 (5)	−0.1425 (2)	0.1201 (19)	
H24'	−0.1773	0.3395	−0.1772	0.144*	
C25	0.3669 (3)	0.6630 (3)	0.3166 (3)	0.0902 (13)	
H25	0.3866	0.7260	0.3327	0.108*	
C25'	−0.1948 (3)	0.2307 (5)	−0.1127 (3)	0.122 (2)	
H25'	−0.2602	0.2243	−0.1278	0.147*	
C26	0.3203 (3)	0.6012 (3)	0.3549 (2)	0.0913 (12)	
H26	0.3081	0.6227	0.3967	0.110*	
C26'	−0.1475 (3)	0.1711 (4)	−0.0598 (3)	0.1068 (15)	
H26'	−0.1808	0.1246	−0.0392	0.128*	
C27	0.2920 (2)	0.5070 (2)	0.33056 (15)	0.0643 (8)	
H27	0.2609	0.4654	0.3563	0.077*	
C27'	−0.0500 (2)	0.1820 (3)	−0.0381 (2)	0.0796 (10)	
H27'	−0.0185	0.1433	−0.0019	0.095*	

### Atomic displacement parameters (Å^2^)

**Table:**

	*U*^11^	*U*^22^	*U*^33^	*U*^12^	*U*^13^	*U*^23^
O1	0.0416 (11)	0.0534 (12)	0.0663 (12)	−0.0047 (9)	0.0033 (9)	0.0017 (10)
O1'	0.0679 (14)	0.0671 (15)	0.1050 (18)	−0.0086 (12)	0.0112 (12)	−0.0422 (14)
C1	0.0422 (15)	0.0393 (14)	0.0415 (13)	−0.0041 (11)	0.0070 (11)	0.0061 (11)
C1'	0.0504 (16)	0.0584 (19)	0.0589 (17)	−0.0060 (14)	0.0153 (13)	−0.0190 (15)
C2	0.0439 (14)	0.0398 (14)	0.0457 (14)	−0.0009 (12)	0.0139 (11)	−0.0013 (12)
C2'	0.0501 (15)	0.0422 (15)	0.0458 (14)	0.0008 (12)	0.0113 (12)	−0.0050 (12)
C3	0.0388 (13)	0.0362 (13)	0.0424 (13)	−0.0013 (11)	0.0103 (10)	−0.0029 (11)
C3'	0.0422 (14)	0.0370 (13)	0.0418 (13)	−0.0014 (11)	0.0093 (11)	−0.0041 (11)
C4	0.0438 (14)	0.0372 (14)	0.0391 (13)	0.0001 (11)	0.0085 (10)	−0.0005 (11)
C4'	0.0438 (14)	0.0475 (15)	0.0417 (13)	0.0018 (12)	0.0077 (11)	−0.0050 (12)
C5	0.0430 (13)	0.0402 (14)	0.0404 (13)	−0.0050 (11)	0.0113 (11)	−0.0011 (11)
C5'	0.0537 (16)	0.0622 (19)	0.0471 (15)	−0.0011 (14)	0.0054 (12)	−0.0112 (14)
C6	0.0451 (14)	0.0334 (13)	0.0458 (14)	−0.0012 (11)	0.0025 (11)	−0.0004 (11)
C6'	0.0472 (15)	0.0460 (15)	0.0459 (14)	0.0026 (12)	0.0100 (12)	−0.0120 (13)
C7	0.0517 (17)	0.0484 (17)	0.075 (2)	0.0056 (14)	0.0110 (14)	0.0065 (15)
C7'	0.0641 (19)	0.0612 (19)	0.071 (2)	0.0147 (16)	0.0173 (16)	−0.0021 (16)
C8	0.070 (2)	0.0497 (19)	0.112 (3)	0.0177 (17)	0.014 (2)	0.018 (2)
C8'	0.062 (2)	0.077 (2)	0.105 (3)	0.0181 (19)	0.023 (2)	−0.007 (2)
C9	0.074 (2)	0.055 (2)	0.097 (3)	−0.0004 (18)	0.009 (2)	0.0269 (19)
C9'	0.061 (2)	0.085 (3)	0.091 (3)	−0.0031 (19)	0.0328 (19)	−0.018 (2)
C10	0.0663 (19)	0.0526 (18)	0.0603 (18)	−0.0105 (16)	0.0094 (15)	0.0125 (15)
C10'	0.0635 (19)	0.077 (2)	0.0558 (17)	−0.0107 (18)	0.0203 (15)	−0.0122 (16)
C11	0.0588 (17)	0.0430 (15)	0.0518 (16)	−0.0023 (13)	0.0121 (13)	0.0050 (13)
C11'	0.0514 (16)	0.0658 (19)	0.0474 (15)	0.0002 (14)	0.0095 (13)	−0.0074 (14)
C12	0.0431 (14)	0.0419 (14)	0.0442 (14)	−0.0050 (12)	0.0083 (11)	0.0044 (12)
C12'	0.0473 (15)	0.0423 (15)	0.0406 (13)	−0.0064 (12)	0.0131 (11)	−0.0057 (12)
C13	0.0577 (17)	0.0462 (17)	0.0664 (18)	−0.0127 (14)	0.0116 (14)	−0.0028 (14)
C13'	0.0538 (16)	0.0534 (17)	0.0460 (15)	−0.0045 (14)	0.0167 (12)	−0.0005 (13)
C14	0.065 (2)	0.061 (2)	0.081 (2)	−0.0229 (17)	0.0041 (17)	0.0055 (18)
C14'	0.0587 (18)	0.069 (2)	0.0630 (18)	−0.0216 (16)	0.0197 (15)	−0.0025 (17)
C15	0.0510 (18)	0.077 (2)	0.075 (2)	−0.0225 (17)	0.0071 (16)	0.0205 (19)
C15'	0.089 (2)	0.0524 (19)	0.0639 (19)	−0.0241 (18)	0.0311 (17)	−0.0038 (16)
C16	0.0465 (17)	0.103 (3)	0.0613 (19)	−0.0004 (19)	0.0151 (15)	0.013 (2)
C16'	0.115 (3)	0.0438 (18)	0.0616 (19)	−0.0006 (19)	0.033 (2)	0.0073 (16)
C17	0.0518 (18)	0.107 (3)	0.063 (2)	0.019 (2)	0.0195 (15)	−0.002 (2)
C17'	0.109 (3)	0.054 (2)	0.0602 (19)	0.021 (2)	0.0232 (19)	0.0123 (16)
C18	0.0530 (17)	0.070 (2)	0.0572 (17)	0.0113 (15)	0.0114 (14)	−0.0083 (15)
C18'	0.076 (2)	0.063 (2)	0.0487 (16)	0.0175 (17)	0.0138 (15)	0.0059 (15)
C19	0.0390 (14)	0.0543 (17)	0.0427 (14)	0.0057 (12)	0.0086 (11)	0.0020 (12)
C19'	0.0604 (17)	0.0467 (16)	0.0372 (13)	0.0046 (13)	0.0131 (12)	−0.0011 (12)
C20	0.0436 (15)	0.074 (2)	0.0522 (16)	−0.0029 (15)	0.0086 (13)	0.0191 (15)
C20'	0.084 (2)	0.0453 (17)	0.0466 (15)	−0.0068 (16)	0.0260 (15)	−0.0025 (13)
C21	0.0368 (13)	0.0523 (16)	0.0457 (14)	−0.0018 (12)	0.0068 (11)	0.0094 (13)
C21'	0.0596 (17)	0.0410 (15)	0.0359 (13)	−0.0038 (13)	0.0173 (12)	−0.0022 (11)
C22	0.0409 (14)	0.0418 (15)	0.0520 (15)	0.0010 (12)	0.0028 (11)	−0.0026 (13)
C22'	0.0517 (17)	0.080 (2)	0.0557 (18)	0.0043 (17)	−0.0005 (14)	−0.0268 (17)
C23	0.069 (2)	0.0473 (17)	0.080 (2)	−0.0124 (15)	0.0178 (17)	−0.0042 (16)
C23'	0.075 (2)	0.133 (4)	0.057 (2)	0.021 (2)	−0.0047 (18)	−0.013 (2)
C24	0.069 (2)	0.0457 (19)	0.126 (3)	−0.0097 (17)	0.006 (2)	0.000 (2)
C24'	0.071 (3)	0.192 (6)	0.082 (3)	0.038 (3)	−0.021 (2)	−0.039 (3)
C25	0.078 (3)	0.044 (2)	0.132 (4)	0.0008 (18)	−0.019 (2)	−0.028 (2)
C25'	0.053 (3)	0.178 (6)	0.128 (4)	0.009 (3)	−0.002 (3)	−0.079 (4)
C26	0.106 (3)	0.074 (3)	0.083 (3)	0.015 (2)	−0.007 (2)	−0.040 (2)
C26'	0.059 (2)	0.115 (4)	0.148 (4)	−0.011 (2)	0.022 (3)	−0.057 (3)
C27	0.073 (2)	0.061 (2)	0.0580 (18)	0.0023 (16)	0.0109 (15)	−0.0106 (16)
C27'	0.055 (2)	0.087 (3)	0.095 (3)	−0.0116 (19)	0.0107 (18)	−0.028 (2)

### Geometric parameters (Å, º)

**Table:**

O1—C1	1.205 (3)	C13—C14	1.407 (4)
O1'—C1'	1.204 (3)	C13—H13	0.9300
C1—C5	1.518 (3)	C13'—C14'	1.412 (4)
C1—C2	1.528 (3)	C13'—H13'	0.9300
C1'—C5'	1.514 (4)	C14—C15	1.354 (4)
C1'—C2'	1.529 (4)	C14—H14	0.9300
C2—C6	1.521 (3)	C14'—C15'	1.362 (4)
C2—C3	1.573 (3)	C14'—H14'	0.9300
C2—H2	0.9800	C15—C20	1.413 (4)
C2'—C6'	1.515 (3)	C15—H15	0.9300
C2'—C3'	1.567 (3)	C15'—C20'	1.402 (4)
C2'—H2'	0.9800	C15'—H15'	0.9300
C3—C12	1.530 (3)	C16—C17	1.360 (5)
C3—C3'	1.578 (3)	C16—C20	1.405 (4)
C3—C4	1.594 (3)	C16—H16	0.9300
C3'—C12'	1.533 (3)	C16'—C17'	1.358 (5)
C3'—C4'	1.597 (3)	C16'—C20'	1.416 (4)
C4—C19	1.512 (3)	C16'—H16'	0.9300
C4—C5	1.550 (3)	C17—C18	1.412 (4)
C4—H4	0.9800	C17—H17	0.9300
C4'—C19'	1.508 (4)	C17'—C18'	1.405 (5)
C4'—C5'	1.551 (3)	C17'—H17'	0.9300
C4'—H4'	0.9800	C18—C19	1.364 (4)
C5—C22	1.514 (3)	C18—H18	0.9300
C5—H5	0.9800	C18'—C19'	1.370 (4)
C5'—C22'	1.514 (4)	C18'—H18'	0.9300
C5'—H5'	0.9800	C19—C21	1.402 (4)
C6—C7	1.383 (4)	C19'—C21'	1.395 (4)
C6—C11	1.389 (4)	C20—C21	1.412 (4)
C6'—C7'	1.378 (4)	C20'—C21'	1.406 (4)
C6'—C11'	1.388 (4)	C22—C23	1.376 (4)
C7—C8	1.394 (4)	C22—C27	1.383 (4)
C7—H7	0.9300	C22'—C27'	1.375 (5)
C7'—C8'	1.394 (4)	C22'—C23'	1.388 (5)
C7'—H7'	0.9300	C23—C24	1.373 (4)
C8—C9	1.373 (5)	C23—H23	0.9300
C8—H8	0.9300	C23'—C24'	1.385 (5)
C8'—C9'	1.363 (5)	C23'—H23'	0.9300
C8'—H8'	0.9300	C24—C25	1.358 (5)
C9—C10	1.359 (4)	C24—H24	0.9300
C9—H9	0.9300	C24'—C25'	1.363 (7)
C9'—C10'	1.372 (5)	C24'—H24'	0.9300
C9'—H9'	0.9300	C25—C26	1.387 (5)
C10—C11	1.375 (4)	C25—H25	0.9300
C10—H10	0.9300	C25'—C26'	1.388 (7)
C10'—C11'	1.375 (4)	C25'—H25'	0.9300
C10'—H10'	0.9300	C26—C27	1.388 (5)
C11—H11	0.9300	C26—H26	0.9300
C11'—H11'	0.9300	C26'—C27'	1.385 (5)
C12—C13	1.371 (4)	C26'—H26'	0.9300
C12—C21	1.402 (3)	C27—H27	0.9300
C12'—C13'	1.365 (3)	C27'—H27'	0.9300
C12'—C21'	1.407 (3)		
			
O1—C1—C5	126.3 (2)	C13'—C12'—C21'	118.3 (2)
O1—C1—C2	124.3 (2)	C13'—C12'—C3'	132.7 (2)
C5—C1—C2	109.4 (2)	C21'—C12'—C3'	109.0 (2)
O1'—C1'—C5'	126.0 (3)	C12—C13—C14	119.2 (3)
O1'—C1'—C2'	123.5 (3)	C12—C13—H13	120.4
C5'—C1'—C2'	110.4 (2)	C14—C13—H13	120.4
C6—C2—C1	109.19 (19)	C12'—C13'—C14'	119.1 (3)
C6—C2—C3	117.6 (2)	C12'—C13'—H13'	120.4
C1—C2—C3	104.56 (19)	C14'—C13'—H13'	120.4
C6—C2—H2	108.4	C15—C14—C13	122.8 (3)
C1—C2—H2	108.4	C15—C14—H14	118.6
C3—C2—H2	108.4	C13—C14—H14	118.6
C6'—C2'—C1'	109.3 (2)	C15'—C14'—C13'	122.5 (3)
C6'—C2'—C3'	117.0 (2)	C15'—C14'—H14'	118.8
C1'—C2'—C3'	105.6 (2)	C13'—C14'—H14'	118.8
C6'—C2'—H2'	108.2	C14—C15—C20	120.4 (3)
C1'—C2'—H2'	108.2	C14—C15—H15	119.8
C3'—C2'—H2'	108.2	C20—C15—H15	119.8
C12—C3—C2	114.3 (2)	C14'—C15'—C20'	120.1 (3)
C12—C3—C3'	111.90 (19)	C14'—C15'—H15'	119.9
C2—C3—C3'	110.57 (18)	C20'—C15'—H15'	119.9
C12—C3—C4	103.32 (18)	C17—C16—C20	120.2 (3)
C2—C3—C4	105.72 (18)	C17—C16—H16	119.9
C3'—C3—C4	110.64 (19)	C20—C16—H16	119.9
C12'—C3'—C2'	114.3 (2)	C17'—C16'—C20'	120.6 (3)
C12'—C3'—C3	111.93 (19)	C17'—C16'—H16'	119.7
C2'—C3'—C3	110.7 (2)	C20'—C16'—H16'	119.7
C12'—C3'—C4'	103.0 (2)	C16—C17—C18	122.6 (3)
C2'—C3'—C4'	105.83 (19)	C16—C17—H17	118.7
C3—C3'—C4'	110.55 (19)	C18—C17—H17	118.7
C19—C4—C5	113.7 (2)	C16'—C17'—C18'	122.6 (3)
C19—C4—C3	105.17 (19)	C16'—C17'—H17'	118.7
C5—C4—C3	107.35 (19)	C18'—C17'—H17'	118.7
C19—C4—H4	110.2	C19—C18—C17	118.7 (3)
C5—C4—H4	110.2	C19—C18—H18	120.7
C3—C4—H4	110.2	C17—C18—H18	120.7
C19'—C4'—C5'	113.8 (2)	C19'—C18'—C17'	118.5 (3)
C19'—C4'—C3'	105.4 (2)	C19'—C18'—H18'	120.8
C5'—C4'—C3'	107.8 (2)	C17'—C18'—H18'	120.8
C19'—C4'—H4'	109.9	C18—C19—C21	119.2 (2)
C5'—C4'—H4'	109.9	C18—C19—C4	132.1 (3)
C3'—C4'—H4'	109.9	C21—C19—C4	108.6 (2)
C22—C5—C1	115.3 (2)	C18'—C19'—C21'	119.3 (3)
C22—C5—C4	116.9 (2)	C18'—C19'—C4'	131.8 (3)
C1—C5—C4	103.97 (19)	C21'—C19'—C4'	108.9 (2)
C22—C5—H5	106.7	C16—C20—C21	116.7 (3)
C1—C5—H5	106.7	C16—C20—C15	127.5 (3)
C4—C5—H5	106.7	C21—C20—C15	115.9 (3)
C1'—C5'—C22'	116.7 (3)	C15'—C20'—C21'	116.7 (3)
C1'—C5'—C4'	105.0 (2)	C15'—C20'—C16'	127.4 (3)
C22'—C5'—C4'	115.4 (2)	C21'—C20'—C16'	115.9 (3)
C1'—C5'—H5'	106.3	C19—C21—C12	113.6 (2)
C22'—C5'—H5'	106.3	C19—C21—C20	122.6 (3)
C4'—C5'—H5'	106.3	C12—C21—C20	123.7 (3)
C7—C6—C11	117.6 (2)	C19'—C21'—C20'	123.2 (3)
C7—C6—C2	118.8 (2)	C19'—C21'—C12'	113.6 (2)
C11—C6—C2	123.5 (2)	C20'—C21'—C12'	123.2 (3)
C7'—C6'—C11'	117.6 (3)	C23—C22—C27	118.4 (3)
C7'—C6'—C2'	119.9 (3)	C23—C22—C5	121.5 (2)
C11'—C6'—C2'	122.5 (2)	C27—C22—C5	120.2 (2)
C6—C7—C8	120.7 (3)	C27'—C22'—C23'	118.3 (3)
C6—C7—H7	119.7	C27'—C22'—C5'	121.9 (3)
C8—C7—H7	119.7	C23'—C22'—C5'	119.7 (3)
C6'—C7'—C8'	120.6 (3)	C24—C23—C22	121.2 (3)
C6'—C7'—H7'	119.7	C24—C23—H23	119.4
C8'—C7'—H7'	119.7	C22—C23—H23	119.4
C9—C8—C7	119.9 (3)	C24'—C23'—C22'	120.5 (4)
C9—C8—H8	120.0	C24'—C23'—H23'	119.8
C7—C8—H8	120.0	C22'—C23'—H23'	119.8
C9'—C8'—C7'	120.9 (3)	C25—C24—C23	120.6 (4)
C9'—C8'—H8'	119.6	C25—C24—H24	119.7
C7'—C8'—H8'	119.6	C23—C24—H24	119.7
C10—C9—C8	120.1 (3)	C25'—C24'—C23'	120.5 (5)
C10—C9—H9	120.0	C25'—C24'—H24'	119.7
C8—C9—H9	120.0	C23'—C24'—H24'	119.7
C8'—C9'—C10'	119.1 (3)	C24—C25—C26	119.6 (3)
C8'—C9'—H9'	120.4	C24—C25—H25	120.2
C10'—C9'—H9'	120.4	C26—C25—H25	120.2
C9—C10—C11	120.2 (3)	C24'—C25'—C26'	120.0 (4)
C9—C10—H10	119.9	C24'—C25'—H25'	120.0
C11—C10—H10	119.9	C26'—C25'—H25'	120.0
C9'—C10'—C11'	120.3 (3)	C25—C26—C27	119.6 (4)
C9'—C10'—H10'	119.9	C25—C26—H26	120.2
C11'—C10'—H10'	119.9	C27—C26—H26	120.2
C10—C11—C6	121.5 (3)	C27'—C26'—C25'	119.1 (5)
C10—C11—H11	119.3	C27'—C26'—H26'	120.5
C6—C11—H11	119.3	C25'—C26'—H26'	120.5
C10'—C11'—C6'	121.6 (3)	C22—C27—C26	120.6 (3)
C10'—C11'—H11'	119.2	C22—C27—H27	119.7
C6'—C11'—H11'	119.2	C26—C27—H27	119.7
C13—C12—C21	118.0 (2)	C22'—C27'—C26'	121.6 (4)
C13—C12—C3	132.9 (2)	C22'—C27'—H27'	119.2
C21—C12—C3	109.0 (2)	C26'—C27'—H27'	119.2
			
O1—C1—C2—C6	80.6 (3)	C2'—C3'—C12'—C13'	−60.7 (4)
C5—C1—C2—C6	−98.4 (2)	C3—C3'—C12'—C13'	66.2 (3)
O1—C1—C2—C3	−152.8 (2)	C4'—C3'—C12'—C13'	−175.0 (3)
C5—C1—C2—C3	28.3 (2)	C2'—C3'—C12'—C21'	118.3 (2)
O1'—C1'—C2'—C6'	73.5 (4)	C3—C3'—C12'—C21'	−114.8 (2)
C5'—C1'—C2'—C6'	−104.6 (3)	C4'—C3'—C12'—C21'	4.0 (2)
O1'—C1'—C2'—C3'	−159.8 (3)	C21—C12—C13—C14	2.8 (4)
C5'—C1'—C2'—C3'	22.1 (3)	C3—C12—C13—C14	179.9 (3)
C6—C2—C3—C12	−5.6 (3)	C21'—C12'—C13'—C14'	0.8 (4)
C1—C2—C3—C12	−126.8 (2)	C3'—C12'—C13'—C14'	179.8 (2)
C6—C2—C3—C3'	−132.9 (2)	C12—C13—C14—C15	−1.3 (5)
C1—C2—C3—C3'	105.9 (2)	C12'—C13'—C14'—C15'	0.0 (4)
C6—C2—C3—C4	107.3 (2)	C13—C14—C15—C20	−1.1 (5)
C1—C2—C3—C4	−13.9 (2)	C13'—C14'—C15'—C20'	−1.0 (5)
C6'—C2'—C3'—C12'	−1.7 (3)	C20—C16—C17—C18	1.4 (5)
C1'—C2'—C3'—C12'	−123.6 (2)	C20'—C16'—C17'—C18'	−0.5 (5)
C6'—C2'—C3'—C3	−129.3 (2)	C16—C17—C18—C19	−0.6 (5)
C1'—C2'—C3'—C3	108.9 (2)	C16'—C17'—C18'—C19'	1.0 (5)
C6'—C2'—C3'—C4'	110.9 (2)	C17—C18—C19—C21	0.2 (4)
C1'—C2'—C3'—C4'	−11.0 (3)	C17—C18—C19—C4	−175.8 (3)
C12—C3—C3'—C12'	165.0 (2)	C5—C4—C19—C18	63.3 (4)
C2—C3—C3'—C12'	−66.4 (2)	C3—C4—C19—C18	−179.5 (3)
C4—C3—C3'—C12'	50.4 (3)	C5—C4—C19—C21	−113.0 (2)
C12—C3—C3'—C2'	−66.2 (2)	C3—C4—C19—C21	4.2 (3)
C2—C3—C3'—C2'	62.4 (2)	C17'—C18'—C19'—C21'	−0.2 (4)
C4—C3—C3'—C2'	179.22 (19)	C17'—C18'—C19'—C4'	−177.5 (3)
C12—C3—C3'—C4'	50.8 (3)	C5'—C4'—C19'—C18'	63.4 (4)
C2—C3—C3'—C4'	179.41 (19)	C3'—C4'—C19'—C18'	−178.8 (3)
C4—C3—C3'—C4'	−63.8 (2)	C5'—C4'—C19'—C21'	−114.1 (2)
C12—C3—C4—C19	−5.3 (2)	C3'—C4'—C19'—C21'	3.7 (3)
C2—C3—C4—C19	−125.6 (2)	C17—C16—C20—C21	−1.8 (4)
C3'—C3—C4—C19	114.6 (2)	C17—C16—C20—C15	176.8 (3)
C12—C3—C4—C5	116.1 (2)	C14—C15—C20—C16	−176.9 (3)
C2—C3—C4—C5	−4.3 (2)	C14—C15—C20—C21	1.8 (4)
C3'—C3—C4—C5	−124.0 (2)	C14'—C15'—C20'—C21'	1.1 (4)
C12'—C3'—C4'—C19'	−4.5 (2)	C14'—C15'—C20'—C16'	−178.5 (3)
C2'—C3'—C4'—C19'	−124.8 (2)	C17'—C16'—C20'—C15'	178.8 (3)
C3—C3'—C4'—C19'	115.2 (2)	C17'—C16'—C20'—C21'	−0.8 (4)
C12'—C3'—C4'—C5'	117.2 (2)	C18—C19—C21—C12	−178.1 (2)
C2'—C3'—C4'—C5'	−3.0 (3)	C4—C19—C21—C12	−1.2 (3)
C3—C3'—C4'—C5'	−123.0 (2)	C18—C19—C21—C20	−0.7 (4)
O1—C1—C5—C22	20.9 (4)	C4—C19—C21—C20	176.2 (2)
C2—C1—C5—C22	−160.2 (2)	C13—C12—C21—C19	175.2 (2)
O1—C1—C5—C4	150.2 (2)	C3—C12—C21—C19	−2.5 (3)
C2—C1—C5—C4	−30.9 (2)	C13—C12—C21—C20	−2.1 (4)
C19—C4—C5—C22	−95.0 (3)	C3—C12—C21—C20	−179.8 (2)
C3—C4—C5—C22	149.1 (2)	C16—C20—C21—C19	1.5 (4)
C19—C4—C5—C1	136.7 (2)	C15—C20—C21—C19	−177.3 (2)
C3—C4—C5—C1	20.8 (2)	C16—C20—C21—C12	178.6 (2)
O1'—C1'—C5'—C22'	29.0 (4)	C15—C20—C21—C12	−0.2 (4)
C2'—C1'—C5'—C22'	−153.0 (2)	C18'—C19'—C21'—C20'	−1.2 (4)
O1'—C1'—C5'—C4'	158.1 (3)	C4'—C19'—C21'—C20'	176.7 (2)
C2'—C1'—C5'—C4'	−23.9 (3)	C18'—C19'—C21'—C12'	−179.1 (2)
C19'—C4'—C5'—C1'	132.4 (2)	C4'—C19'—C21'—C12'	−1.2 (3)
C3'—C4'—C5'—C1'	16.0 (3)	C15'—C20'—C21'—C19'	−178.0 (2)
C19'—C4'—C5'—C22'	−97.7 (3)	C16'—C20'—C21'—C19'	1.7 (4)
C3'—C4'—C5'—C22'	145.9 (3)	C15'—C20'—C21'—C12'	−0.3 (4)
C1—C2—C6—C7	−113.7 (3)	C16'—C20'—C21'—C12'	179.4 (2)
C3—C2—C6—C7	127.4 (3)	C13'—C12'—C21'—C19'	177.2 (2)
C1—C2—C6—C11	64.2 (3)	C3'—C12'—C21'—C19'	−2.0 (3)
C3—C2—C6—C11	−54.7 (3)	C13'—C12'—C21'—C20'	−0.7 (4)
C1'—C2'—C6'—C7'	−123.1 (3)	C3'—C12'—C21'—C20'	−179.8 (2)
C3'—C2'—C6'—C7'	117.0 (3)	C1—C5—C22—C23	57.1 (3)
C1'—C2'—C6'—C11'	56.2 (3)	C4—C5—C22—C23	−65.6 (3)
C3'—C2'—C6'—C11'	−63.7 (3)	C1—C5—C22—C27	−122.0 (3)
C11—C6—C7—C8	1.6 (4)	C4—C5—C22—C27	115.4 (3)
C2—C6—C7—C8	179.6 (3)	C1'—C5'—C22'—C27'	42.8 (4)
C11'—C6'—C7'—C8'	0.1 (4)	C4'—C5'—C22'—C27'	−81.2 (4)
C2'—C6'—C7'—C8'	179.5 (3)	C1'—C5'—C22'—C23'	−139.5 (3)
C6—C7—C8—C9	−1.3 (5)	C4'—C5'—C22'—C23'	96.5 (3)
C6'—C7'—C8'—C9'	−0.5 (5)	C27—C22—C23—C24	0.8 (4)
C7—C8—C9—C10	0.3 (5)	C5—C22—C23—C24	−178.3 (3)
C7'—C8'—C9'—C10'	0.7 (5)	C27'—C22'—C23'—C24'	0.4 (5)
C8—C9—C10—C11	0.3 (5)	C5'—C22'—C23'—C24'	−177.4 (3)
C8'—C9'—C10'—C11'	−0.7 (5)	C22—C23—C24—C25	−0.6 (5)
C9—C10—C11—C6	0.1 (4)	C22'—C23'—C24'—C25'	−1.9 (6)
C7—C6—C11—C10	−1.0 (4)	C23—C24—C25—C26	−0.1 (6)
C2—C6—C11—C10	−178.9 (2)	C23'—C24'—C25'—C26'	1.6 (7)
C9'—C10'—C11'—C6'	0.3 (4)	C24—C25—C26—C27	0.5 (6)
C7'—C6'—C11'—C10'	−0.1 (4)	C24'—C25'—C26'—C27'	0.1 (7)
C2'—C6'—C11'—C10'	−179.4 (2)	C23—C22—C27—C26	−0.4 (4)
C2—C3—C12—C13	−58.1 (4)	C5—C22—C27—C26	178.7 (3)
C3'—C3—C12—C13	68.5 (4)	C25—C26—C27—C22	−0.2 (5)
C4—C3—C12—C13	−172.4 (3)	C23'—C22'—C27'—C26'	1.3 (5)
C2—C3—C12—C21	119.1 (2)	C5'—C22'—C27'—C26'	179.1 (3)
C3'—C3—C12—C21	−114.2 (2)	C25'—C26'—C27'—C22'	−1.6 (6)
C4—C3—C12—C21	4.8 (3)		
